# Body Shape and Life Style of the Extinct Balearic Dormouse *Hypnomys* (Rodentia, Gliridae): New Evidence from the Study of Associated Skeletons

**DOI:** 10.1371/journal.pone.0015817

**Published:** 2010-12-31

**Authors:** Pere Bover, Josep A. Alcover, Jacques J. Michaux, Lionel Hautier, Rainer Hutterer

**Affiliations:** 1 Departament de Biodiversitat i Conservació, Institut Mediterrani d'Estudis Avançats, Esporles, Spain; 2 Division of Vertebrate Zoology/Mammalogy, American Museum of Natural History, New York, New York, United States of America; 3 EPHE and ISEM, UMR 5554 CNRS Université Montpellier II and Université de Montpellier, Montpellier, France; 4 Department of Zoology, University of Cambridge, Cambridge, United Kingdom; 5 Zoologisches Forschungsmuseum Alexander Koenig, Bonn, Germany; University College London, United Kingdom

## Abstract

*Hypnomys* is a genus of Gliridae (Rodentia) that occurred in the Balearic Islands until Late Holocene. Recent finding of a complete skeleton of the chronospecies *H. morpheus* (Late Pleistocene-Early Holocene) and two articulated skeletons of *H.* cf. *onicensis* (Late Pliocene) allowed the inference of body size and the calculation of several postcranial indexes. We also performed a Factorial Discriminant Analysis (FDA) in order to evaluate locomotory behaviour and body shape of the taxa. Using allometric models based on skull and tooth measurements, we calculated a body weight between 173 and 284 g for *H. morpheus*, and direct measurements of articulated skeletons yielded a Head and Body Length (HBL) of 179 mm and a Total Body Length of 295 mm for this species. In addition to the generally higher robustness of postcranial bones already recorded by previous authors, *H. morpheus,* similar to *Canariomys tamarani,* another extinct island species, displayed elongated zygopodium bones of the limbs and a wider distal humerus and femur than in an extant related taxon, *Eliomys quercinus*. Indexes indicated that *Hypnomys* was more terrestrial and had greater fossorial abilities than *E. quercinus*. This was also corroborated by a Discriminant Analysis, although no clear additional inference of locomotory abilities could be calculated.

## Introduction

**Figure 1:**
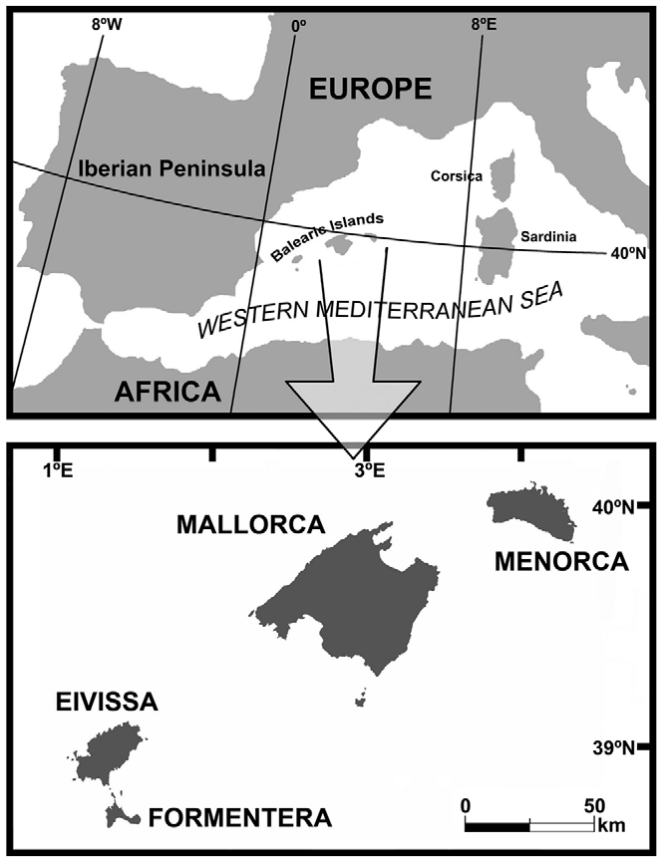
Geographical location of the Balearic Islands.

The autochthonous mammal fauna of the upper Pleistocene and Holocene of Mallorca and Menorca (Western Mediterranean, Figure 1) consists of three endemic species. Remains of *Myotragus balearicus* (Artiodactyla: Bovidae), *Hypnomys morpheus* (Rodentia: Gliridae) and *Nesiotites hidalgo* (Soricomorpha: Soricidae) are abundant in the fossil sites of these islands. They all derived from a single Messinian colonization event to Mallorca, and then colonized Menorca probably during the first upper Pliocene glaciations. They are the most recent representatives of three lineages that evolved in isolation during the last 5.35 My, and represent the surviving lineages until late Holocene of a wider colonisation stock [1]–[6].

The extinction of these mammals took place during the Holocene, and was probably related to the arrival of humans [7], [8]. The long evolution under isolation (in absence of mammalian carnivores and other herbivores) allowed the emergence of remarkable anatomical and physiological traits. The evolution of *Myotragus* has been accurately tracked through an impressive fossil record documenting (1) decrease in size, (2) brain reduction, (3) changes related to locomotion, and (4) reduction in the number of incisors and premolars. *M. balearicus*, the most recent species of its phylum, with a shoulder height of c. 50 cm, displayed shortened legs, a single ever-growing incisor in each jaw, one lower premolar and two upper premolars. The studies on the evolution of the other two mammals, *Hypnomys* and *Nesiotites*, focused mainly on differences in tooth size and anatomy [9]. In this paper we approach some aspects of the *Hypnomys* evolution by way of the reconstruction of its body size and shape.

Reconstruction of extinct rodents is a difficult task as they are mainly documented by teeth and isolated bones. The finding of complete or nearly complete skeletons is consequently very important. Life aspect of extinct mammals can be highly informative to assess their morphology, behaviour and ecology. Two components of a species' life history are available from associated material, the body size and the proportions of different parts of the skeleton as the skull and the limbs. Body design and size of mammalian species are relevant characteristics for the analysis of their morphology and ecology, as many traits of physiology and life history scale with size [10]. The body parameters are easily obtained in extant species, but different methods should be applied in order to approach the body size and, to a lesser degree, body shape for extinct species from their bones. There is an abundant literature on the subject [11]–[15]. In rodents, despite the large fossil record, the lack of postcranial identifiable bones and the difficulties to establish relationship between cranial and postcranial remains in multi-species deposits, frequently limit the weight estimates to those derived from the teeth and other cranial parameters, while they constrain the approach to other body parameters.

Several proxies have been proposed to infer body weight in extinct rodents. All these methods are related to measurements of different parts of the skeleton: postcranial bones [15]–[17], skull [18], isolated incisors [19], [20] or isolated molars [21], [22].

No attempts to infer body weight of the fossil dormouse *Hypnomys* (Gliridae) [23] have been published, although Mills (1976) [24] accurately described its skeleton. It was a large dormouse, adapted to eat harder food than *Eliomys quercinus* [25]. Until now, only one skeleton of *Hypnomys morpheus* has been reported (Cova des Penyal Blanc, Cabrera)[26]. Unfortunately, it was disarticulated and covered by a thick flowstone layer, precluding the possibility to measure the bones and to estimate its body size and proportions. Recently collected associated material of this species offers a unique opportunity to estimate its body size parameters, and allows a reliable approach to reconstruct its life aspect. In addition, some complete skeletons of *Hypnomys* cf. *onicensis,* an ancestor of *H. morpheus* dated c. 2.5-2 My, have been found, some of them embedded in flowstone and with bones in articulation. The goal of this paper is to present the body shape and weight of *Hypnomys* on the basis of this new material.

## Materials and Methods

**Figure 2:**
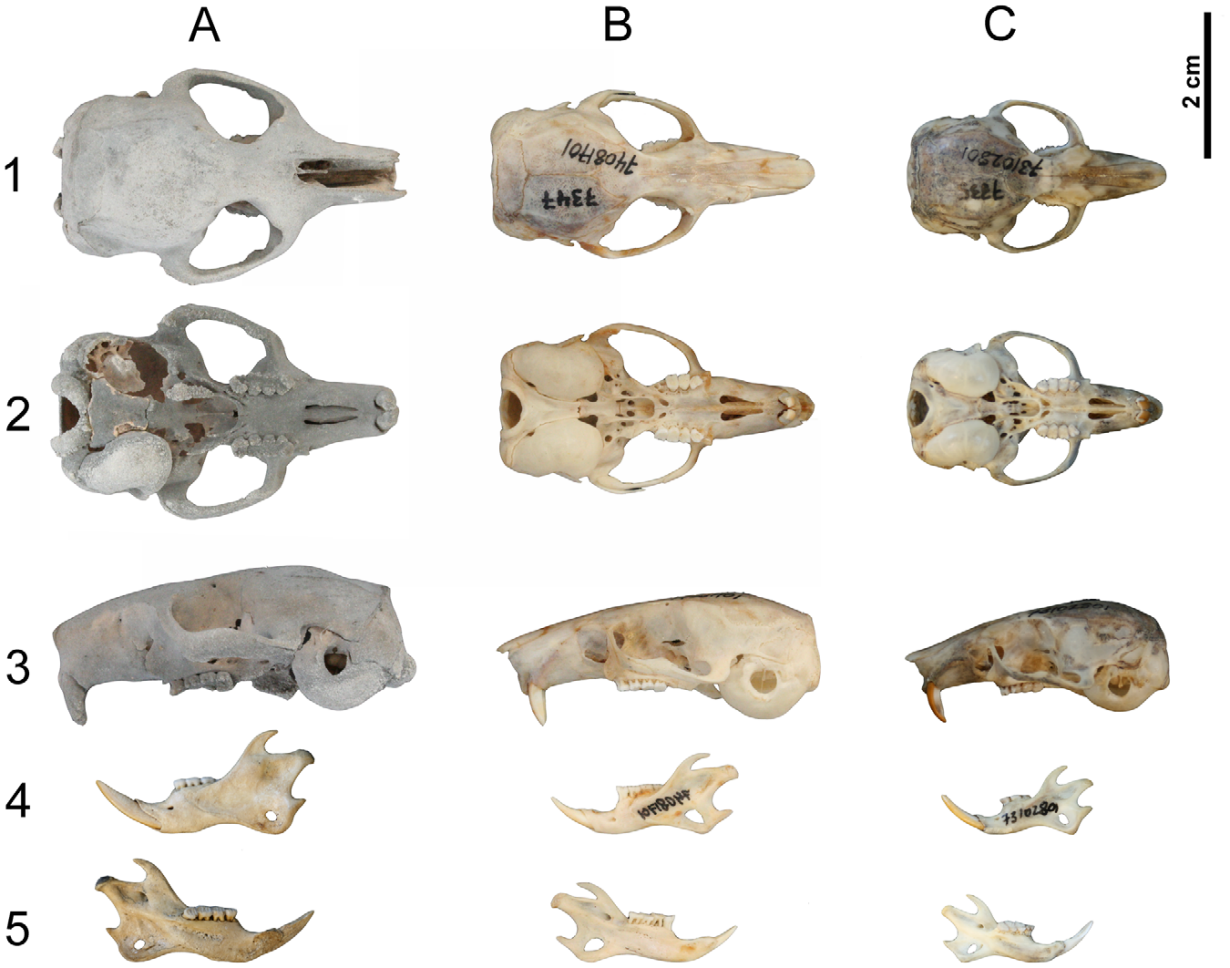
Skull and mandible of glirids considered in this study. A) *Hypnomys morpheus* (CSC-2); B) *Eliomys quecinus ophiusae* (IMEDEA 7347); C) *Eliomys quercinus* s.l. (IMEDEA 7335). Skull in 1) dorsal, 2) ventral and 3) lateral views. Mandible in 4) labial and 5) lingual views.

**Figure 3:**
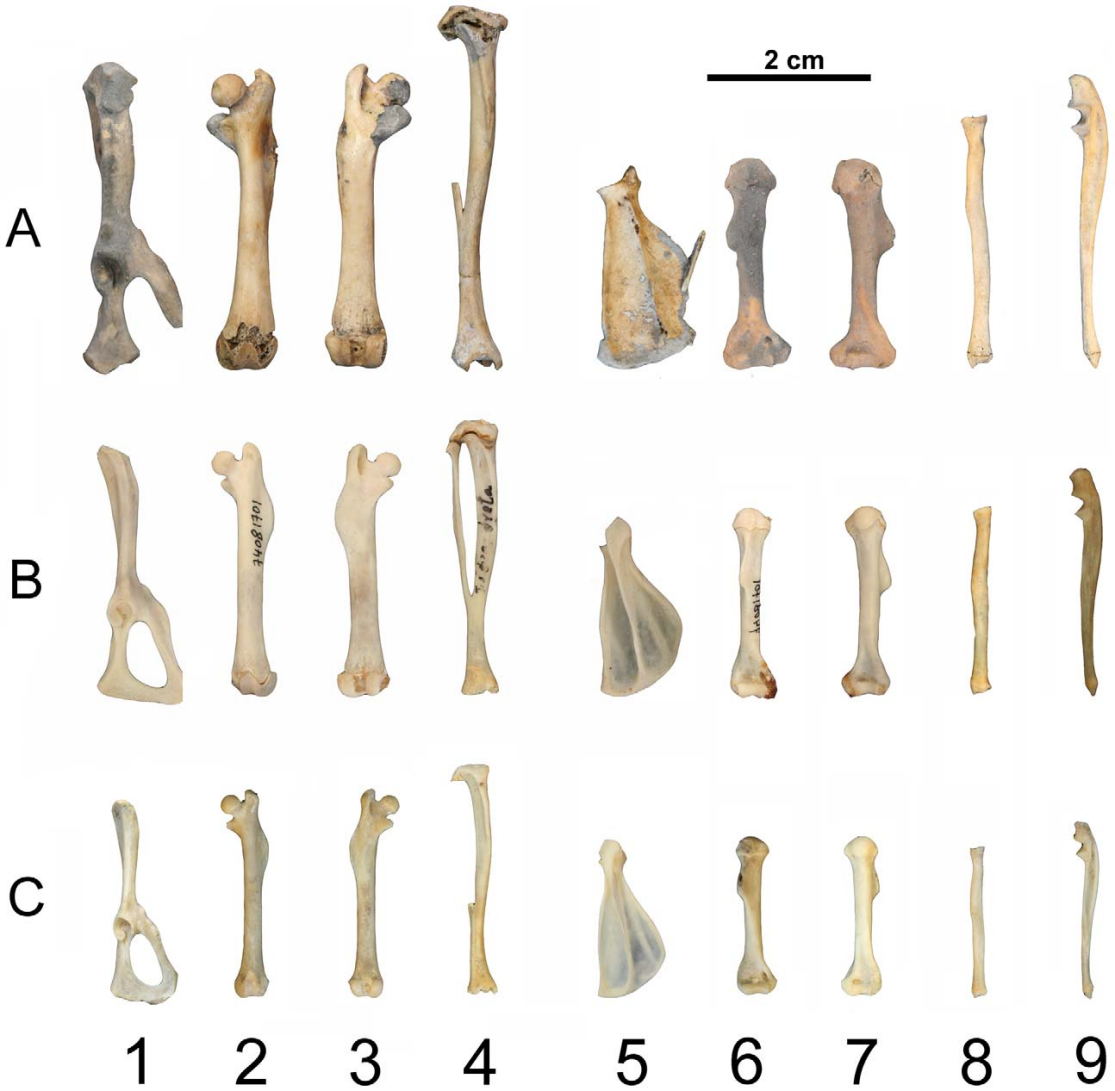
Postcranial bones of the three taxa of glirids considered. A) *Hypnomys morpheus* (CSC-2); B) *Eliomys quecinus ophiusae* (IMEDEA 7347); C) *Eliomys quercinus* s.l. (IMEDEA 7335). 1) Pelvis, lateral view; 2) Femur, cranial view; 3) Femur, caudal view; 4) Tibia, cranial view; 5) Scapula, lateral view; 6) Humerus, caudal view; 7) Humerus, cranial view; 8) Radius, cranial view; 9) Ulna, lateral view.

An almost complete skeleton of *Hypnomys morpheus* (Figures 2a and 3a) was recently discovered in a cave in the northern Mallorcan mountains (Cova des Coral·loides, Calvià, southwest of Serra de Tramuntana)[27]. Its accession number is CDC-2. Other five partial associated skeletons of *H. morpheus* obtained in this cave have been also studied (CDC-10, CDC-27, CDC-39 and CDC-40).

**Figure 4:**
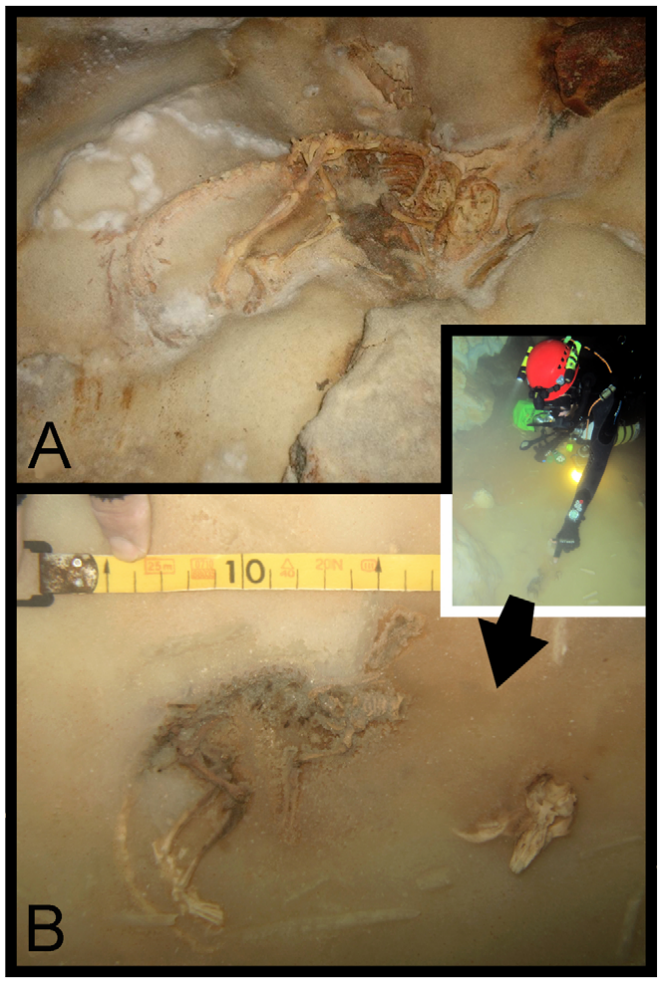
Articulated skeletons of *Hypnomys morpheus* covered by flownstone found in the Cova des Pas de Vallgornera (Llucmajor, Mallorca). A) Skeleton located in a dry passage of the cave. B) Skeleton found in the bottom of a lake (see diver in insert). Photos by G. Mulet (a) and M.A. Perelló (b).

Pictures of different articulated *Hypnomys* cf. *onicensis* skeletons obtained at Cova des Pas de Vallgornera (Llucmajor, Mallorca) [28] were used to obtain direct body measurements (Figure 4). The material was not extracted, particularly because of the difficulties to reach the site and the extreme fragility of the specimens.

**Figure 5:**
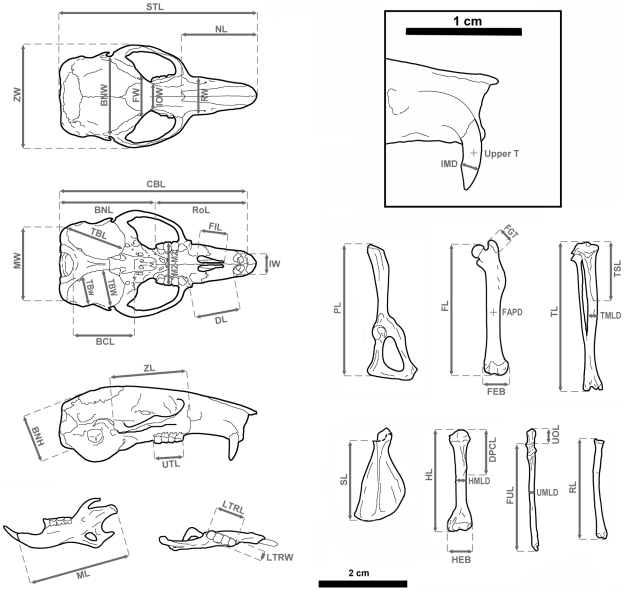
Measurements taken in skull and postcranial bones to calculate the different indexes. See “Material and methods” section for measurement abbreviations.

We compared *Hypnomys* in detail with its closest living relative, *Eliomys quercinus* [23], [29]. Specimens of three populations of *Eliomys quercinus* living in the Balearic Islands (*E. q. quercinus* from Mallorca, *E. q. gymnesicus* from Menorca and *E. q. ophiusae* from Formentera) were included. The two first populations include specimens with a body size similar to the Iberian mainland populations [30], [31], while the Formentera population consists of large-sized animals [32]. These specimens were collected at the end of the 70's and their skeletons are currently curated at IMEDEA. Specimens are listed in Appendix S1. Measurements are illustrated in Figure 5 and follow Samuels & Van Valkenburgh (2008)[33] for postcranial bones and Kahmann (1970)[32] for skull and mandible. All measurements were taken with a digital caliper of an accuracy of 0.02 mm. Additional skull biometrical data of *E. quercinus* from the Balearic Islands have been obtained from Alcover (1983)[34]. Data of condylobasal length and body weight of *E. quercinus* from various Mediterranean islands (Sicily, Sardinia, Lipari, Menorca and Formentera) to infer body weight in *H. morpheus* were taken from Kahmann & Lau (1972)[35].

The following measurements were used (Figure 5). *Skull and mandible*: BCL: Basicranial Length; BNH: Braincase Height; BNL: Braincase Length; BNW: Braincase Width; CBL: Condylobasal Length; DL: Diastema Length; FIL: Foramina Incisivi Length; FW: Frontal Width; IMD: mesiodistal diameter of upper incisor at wear level; IOW: Interorbitary Width; IW: Incisors Width; LTRL: Lower Toothrow Length; LTRW: Lower Toothrow Width; M2-M2: Breadth between labial margins of M2; ML: Mandibular Length; MW: Mastoid Width; NL: Nasal Length; RoL: Rostral Length; RTRA: Rectangular Lower Toothrow Area (calculated as LTRL ×LTRW); RW: Rostral Width; STL: Skull Total Length; TBL: Tympanic Bulla Length; TBW: Tympanic Bulla Maximum Width; TBw: Tympanic Bulla Mimimun Width; Upper T: Upper Incisor Transverse Diameter; UTL: Upper Toothrow Length; ZL: Zygomatic Length; ZW: Zygomatic Width. *Postcranial skeleton*: DPCL: Length of Deltopectoral Crest of the Humerus; FAPD: Midshaft Anteroposterior Diameter of the Femur; FEB: Epicondylar Breadth of the Distal Femur; FGT: Height of the Greater Trochanter of the Femur; FL: Femur Length; FUL: Functional Length of the Ulna; HEB: Epicondylar Breadth of the Distal Humerus; HL: Humerus Length; HMLD: Midshaft Mediolateral Diameter of the Humerus; PL: Pelvis Length; RL: Radius Length; SL: Scapula Length; TL: Tibia Length; TMLD: Midshaft Mediolateral Diameter of the Tibia; TSL: Length of Tibial Tuberosity; UMLD: Midshaft Mediolateral Diameter of the Ulna; UOL: Length of the Olecranon Process of the Ulna. Additionally, we used the Head and Body Length (HBL), Tail Length (TaL) and Weight (W) as external body size parameters.

To obtain body weight estimates, slope and intercept values of allometric models used here were obtained from literature in its log or ln transformation of the power function Y = aX^b^ (estimations based on skull measurements obtained from [36], dental measurements from [19], [22], [37] and postcranial elements from [38]). A ln-transformation of an allometric model for body weight estimation from Condylobasal Length was applied to *Eliomys quercinus* data from [35]. Data for comparative analysis of the body proportions and the locomotion were taken from [33].

The choice of the variables to estimate the body weight is important, because the different proxies give a large variety of results. In order to evaluate the reliability of the equations used to approach the body weight of *Hypnomys*, estimates of body size of an *Eliomys quercinus* specimen from Mallorca (IMEDEA 7335) with known body weight (74 g) were calculated. The best approaches of body weight were obtained through the allometric model of [22] for RTRA (Rectangular lower Tooth Row Area), [19] for IMD, [36] for skull CBL and the linear correlation between body size and CBL obtained from [35].

Statistical procedures were performed with Statistica (version 6.0). The morphological variability of extant and extinct compared dormice (i.e., *Hypnomys* and three insular populations of *E. quercinus*) was quantified using Samuels and Van Valkenburgh (2008)[33] procedure based on measurement of morphological (*e.g*. osteological and muscular) characteristics used to compute limb indexes. For each analysis, a set of 13 robustness, morphofunctional and proportion indexes was considered: SMI, BI, HRI, HEB, OLI, URI, CI, GI, FRI, FEB, TRI, TSI and IM (see Appendix S2 for definitions). Manova in association with a test of significance (Wilk's Lambda test) was performed on these indexes in order to assess the effects of life-style. Then, a Factorial Discriminant Analysis (FDA) of shape coordinates was performed in order to maximize discrimination among rodents belonging to different locomotory groups. The analysed dormice (i.e., *Eliomys* and *Hypnomys*) were supplemented with additional data of other rodents [33].

Acronyms used: IMEDEA (Institut Mediterrani d'Estudis Avançats - Mediterranean Institute for Advanced Studies, Mallorca, Spain), CDC (Cova des Coral·loides, Calvià, Mallorca, deposited at the Societat d'Història Natural de les Balears, Mallorca, Spain).

## Results

### Body size

One of two well preserved articulated skeletons of *Hypnomys* cf. *onicensis* in the Cova des Pas de Vallgornera (Llucmajor, Mallorca; e.g., Figure 4)[28] could be measured although partially covered with flowstone (Figure 4b) and placed on the floor of a currently flooded gallery. It had a total length of 247 mm (head and body length measured adding the skull length to the body length from the atlas to the end of the last sacral: 150 mm; tail length along caudal vertebrae: 97 mm). The tail (TaL) was relatively much shorter (c.65% of HBL) than in *Eliomys quercinus* (c.88% of HBL in adult specimens). Assuming that body proportions have not changed substantially along the *Hypnomys* evolution in Mallorca (an unproved assumption), an estimation of body size in the *H. morpheus* from Cova des Coral·loides can be presented on the basis to its CBL. Total length of this specimen was estimated as c. 295 mm (HBL: 179 mm; TaL: 116 mm), an estimate that agrees with the HBL of 180 mm established by Mills (1976)[24].

Slope and intercept values obtained from the different methods which we used to estimate body weight for the specimen CDC-2 of *H. morpheus* from Cova des Coral·loides are shown in Table S1. Using best proxies (according to the previous evaluation of approaches), values between 173 and 284 g for the specimen *Hypnomys morpheus* CDC-2 from Cova des Coral·loides were obtained. Values obtained from tooth-based models gave estimates between 173 and 260 g, while those based on CBL produced estimates of 214 and 284 g. Nevertheless, it has been established that skull and teeth measurements often produce underestimates of body weight [39]. The relation between head and the body shape will be explored later to check the reliability of these approaches.

### Skull

*E. q. ophiusae* had greater values of indexes related to the proportional elongation of the rostral part of the skull (elongated rostrum, nasal, foramina incisivi and diastema), while corresponding values of *H. morpheus* fell in the range of *E. quercinus* from Mallorca and Menorca, except for index RoL/CBL in which the fossil species showed the greatest value. *E. q. ophiusae* and *H. morpheus* had smaller values for relative braincase length (BNL/CBL)(Table S2, Figure 2).

In general, index values involving the rostrum width (*sensu lato*, including zygomatic, interorbitary, rostral and M2-M2 widths) were similar in *H. morpheus* and normal-sized Mallorcan and Menorcan populations of *E. quercinus*, while *E. q. ophiusae* displayed a proportionally narrower zygomatic and interorbital breadth. On the other hand, *H. morpheus* and *E. q. ophiusae* had smaller values of indexes related to proportional width of the braincase part of the skull (mastoid and braincase width) than *E. quercinus* s.l., indicating a proportionally narrower braincase in the two large-sized taxa. Mastoid width of *H. morpheus* was even proportionally smaller than *E. q. ophiusae*. *H. morpheus* and *E. q. ophiusae* showed lower values of index BNH/CBL than in the other *E. quercinus* populations, indicating a proportionally lower braincase in the first two taxa.

According to the indexes TBL/CBL, TBW/CBL, TBw/CBL and TBL/BNL, the tympanic bulla of *H. morpheus* was shorter but proportionally wider than in *E. quercinus*.

Regarding the dentition, the upper tooth row of *H. morpheus* appeared to be slightly more elongated than in *E. quercinus*. This could be related to an adaptation to a more abrasive diet already hypothesized for the fossil species [25].

### Limbs

*Hypnomys morpheus* had robust limbs, with an elongated zygopodium for both fore- and hindlimbs (Tables S3, S4 and S5; Figure 3)[24]. To estimate the body shape design, a series of bone ratios were explored. These ratios were then compared with the corresponding ratios in the populations of *Eliomys quercinus*.

The obtained indexes were split here in (1) robustness indexes, (2) morphofunctional indexes, and (3) proportion indexes to facilitate their interpretation.

#### Robustness indexes: HRI, URI, FRI and TRI

Among *Hypnomys* and all the studied populations of *Eliomys quercinus*, the greatest values for all indexes related to robustness were displayed by *H. morpheus* and *E. q. ophiusae*, with the highest values for stylopodium in *H. morpheus* (Table S3).

In *Hypnomys morpheus* the range of values of humerus robustness, HRI (0.106–0.116) displayed a slight overlap with *E. q. ophiusae* (0.098–0.110). Average values for *E. quercinus* are 0.095 and 0.091 for the Mallorcan and Menorcan populations respectively, and value range of the Menorcan sample was included in the range of the Mallorcan one. The greatest ulna robustness values were those of *E. q. ophiusae* (average value 0.043, range: 0.039–0.049) *Hypnomys* presented an ulna slightly more slender than that of *E. q. ophiusae* (average 0.041, range: 0.038–0.042).

The highest values for the robustness index of the femur (FRI) were displayed by *H. morpheus* (average 0.098, range 0.088–0.112) while the relatively robust *E. q. ophiusae* showed a smaller average value (average 0.082, range 0.078–0.084). *E. q. gymnesicus* presented slender femora, with average values close to 0.071 and a range smaller than in *E. q. ophiusae*. Similarly to the ulna, *E. q. ophiusae* displayed the greatest value for tibia robustness index (TRI) among populations studied (average 0.066, range 0.062–0.068). *H. morpheus* presented more slender tibiae than *E. q. ophiusae* (average 0.06, range 0.059–0.063). No differences in limb bone robustness were observed between the normal sized populations of *E. quercinus*.

#### Morphofunctional indexes: OLI, TSI, SMI, GI, HEB, FEB

In two of the morphofunctional indexes (OLI and TSI) all groups had similar values and no remarkable differences were observed (Table S4). Roughly, both olecranon process and tibial tuberosity were similarly positioned (same proportional distance from proximal extreme) in *Hypnomys* and *Eliomys*. For two other indexes (SMI and GI) *Hypnomys morpheus* had similar average or range values than *E. q. ophiusae*, while both were larger than Mallorcan and Menorcan populations of *E. quercinus.* The ranges of values of *Hypnomys* and *E. q. ophiusae* were larger than the range observed in the populations which individuals have the size of the mainland garden dormice (except for a slight overlap in GI range values of large glirids and *E. q. gymnesicus* from Mallorca). These indexes indicated a proportionally longer deltopectoral crest (SMI) and distal extension of femoral greater trochanter (GI) in *Hypnomys* and *E.q.ophiusae*.

Remarkable differences in indexes, in which epicondylar breadth of the long bone is involved (HEB and FEB), could be observed in the stylopodium of *H. morpheus* and the extant *Eliomys*. *Hypnomys* had greater average and range values than *Eliomys,* documenting a wider medio-lateral distal breadth in the humerus and femur of *H. morpheus*.

#### Proportion indexes BI, CI, IM

*H. morpheus* had higher values of BI and CI than extant *Eliomys* indicating a proportional elongation of the zygopodium bones, i.e., ulna, radius and tibia (Table S5). These higher indexes, together with URI and TRI, should be interpreted as if *Hypnomys* possessed relatively more elongated zygopodes than *E. q. ophiusae*, a peculiar trait of the genus. Differences could also be observed in the IM index, with lower values in *H. morpheus*, indicating that it had proportionally longer hind limbs than *Eliomys*. Compared to *E. quercinus ophiusae*, in *Hypnomys* the increase of the fore limb zygopodium was greater than the increase in the hind limb zygopodium. Limb proportions were similar in all populations of *Eliomys quercinus* studied, independently of individual size.

When limb bone lengths were compared to the CBL (a proxy of overall size) of the same individual (Table S6), remarkable differences appeared between *Hypnomys* and *Eliomys*. Analyses of these ratios allowed to describe how morphology changed along the *Hypnomys* lineage. In agreement with the conclusions of the analyses of the limb indexes, the higher values of the ratios RL/CBL, FUL/CBL and TL/CBL and similar values of the ratios HL/CBL, FL/CBL and SL/CBL reflected the elongation of the zygopodium bones in *Hypnomys.* Additionally, no remarkable differences could be observed between *Hypnomys* and *Eliomys* in the values of two of the morphofunctional indexes studied (OLI, proportional olecranon process length, and TSI, proportional distal extension of the tibial tuberosity), indicating that an elongation of the zygopodium affected the whole bone (ulna and tibia).

#### Statistical approach

**Figure 6:**
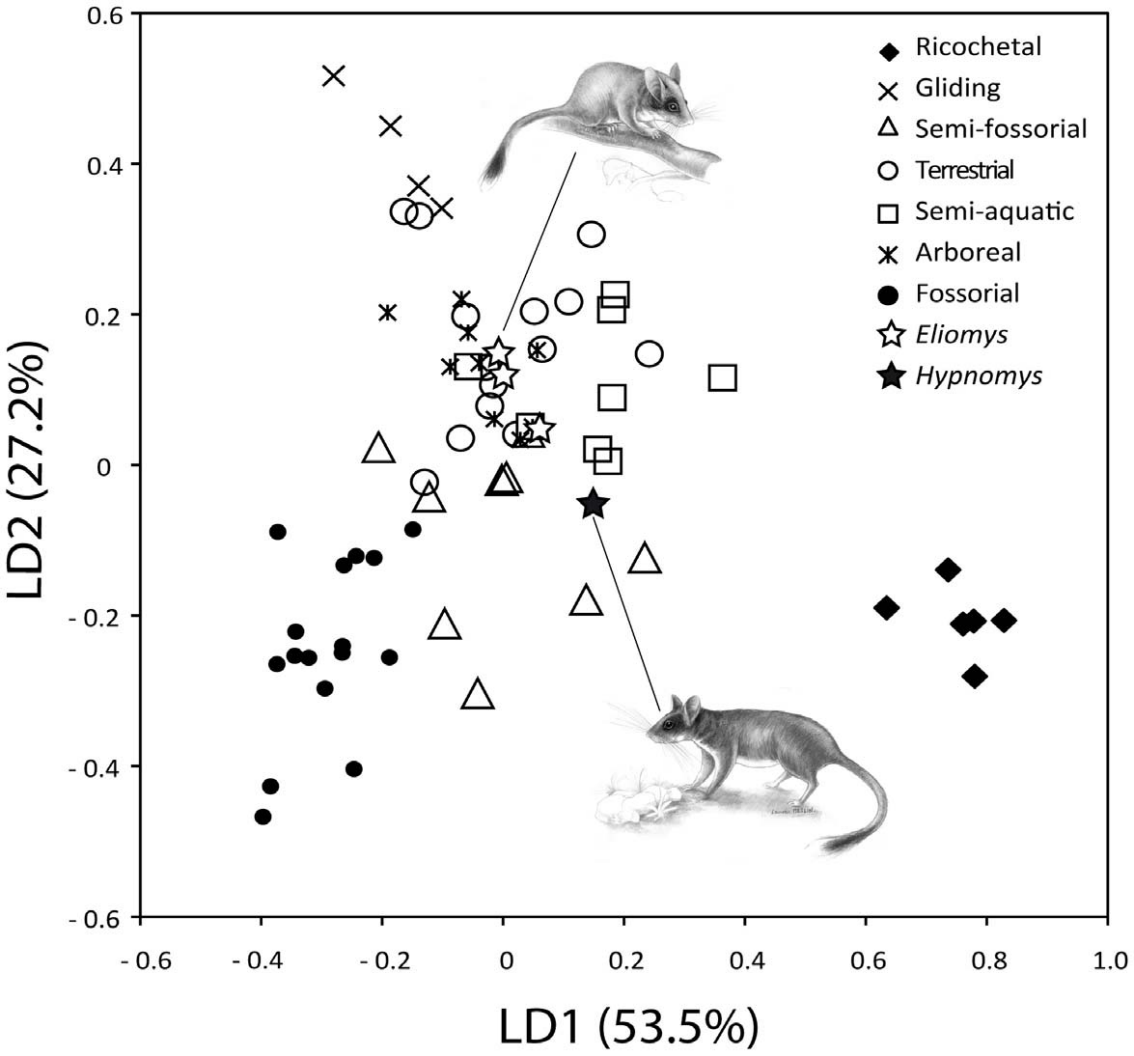
Plot of axes one and two of the factorial discriminant analysis among groups of different locomotor habits.

**Figure 7:**
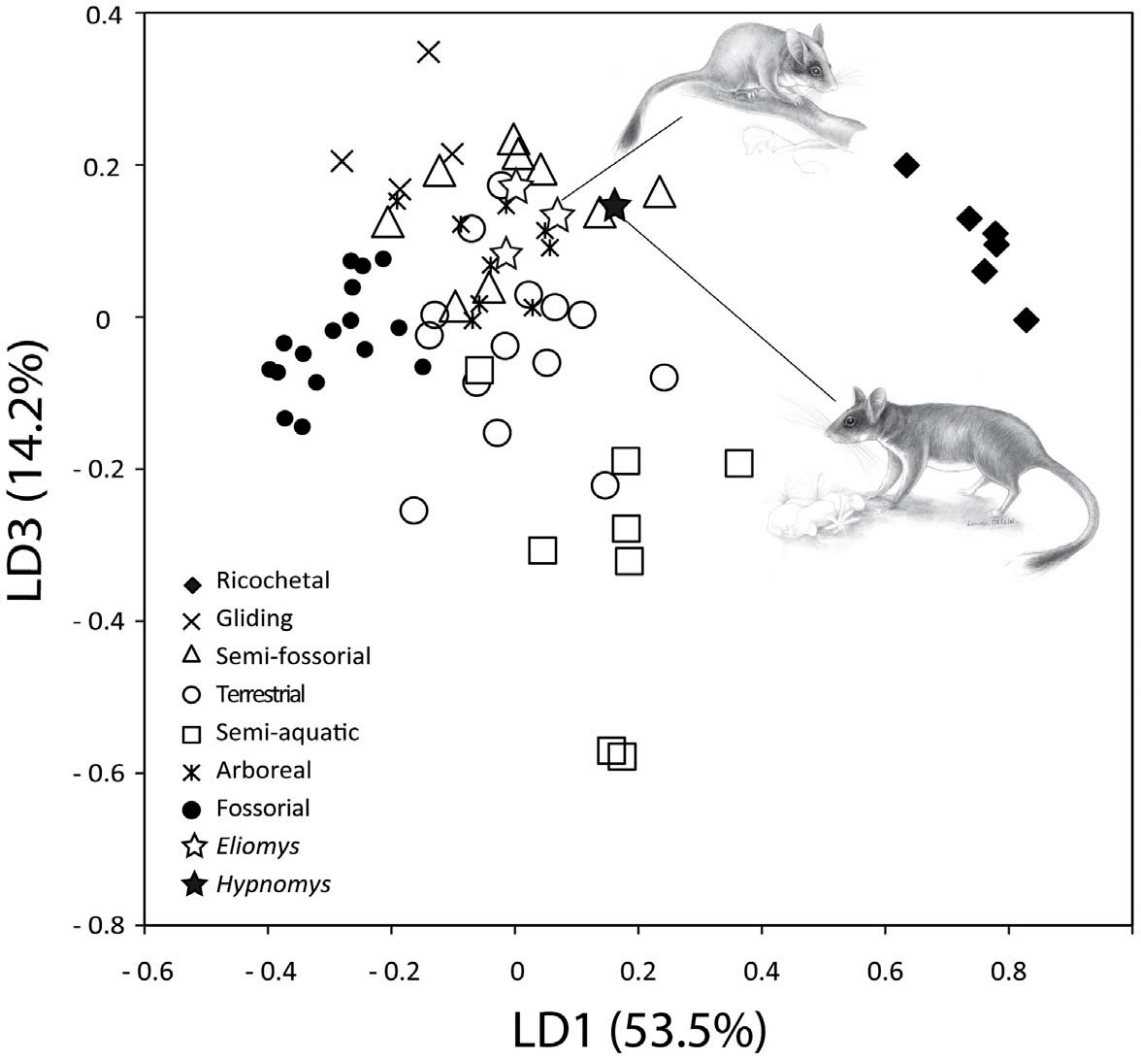
Plot of axes one and three of the factorial discriminant analysis among groups of different locomotor habits.

As Samuels and Van Valkenburgh (2008)[33] previously showed, Manovas on robustness, morphofunctional and proportion indexes indicated a significant differentiation of the morphology within the dataset involving life-style (Wilk's Lambda test: Value = 0.00290, F = 78.265, *p*<0.001). A factorial discriminant analysis, with the addition of extinct taxa as unclassified cases, allowed a complete discrimination among groups of different locomotory habits (Figures 6 and 7). Morphologies in relation with these locomotory habits could be separated on the first three discriminant axes [comprising 95% of among-group variance (Figures 6 and 7)].

The first discriminant axis (LD1) accounted for 53.5% of variance and was positively correlated with brachial index (BI) and crurial index (CI), and negatively correlated with intermembral index (IM). Fossorial, gliding and arboreal rodents tended to have negative scores for LD1 whereas ricochetal rodents showed strongly positive DF1 scores (Figure 6). On the other hand, 27.2% of among-group variance is explained by the second discriminant axis (LD2). This axis was negatively correlated with shoulder moment index (SMI), humeral robustness index (HRI), humeral epicondylar index (HEB), ulnar robustness index (URI), and olecranon length index (OLI). LD2 distinguished gliding and fossorial taxa that tended to have positive and negative sores for this axis. In the shape space defined by LD1 and LD2, the location of Balearic *Eliomys* remained close to arboreal as well as terrestrial rodents whereas *Hypnomys* was located near to semifossorial rodents. The third axis (LD3 - 14.2% of among-group variance - Figure 6) included components positively related to femoral robustness index (FRI), femoral epicondylar index (FEB) and tibial spine index (TSI), and mainly separated semi-aquatic rodents from all other groups. The morphological characteristics of *Hypnomys* and *Eliomys* were less discriminated on the third discriminant axis.

The discriminant model used to separate the locomotor groups was primarily checked using a classification phase and then used on extinct taxa to assess their locomotor habit. This classification showed 89.4% correct classification of species. Fossorial, gliding and ricochetal groups had 100% correct classification. All other groups had <90% correct classification but >75%: 88.9% for semifossorial, 87.5% for semiaquatic, 78.6% for terrestrial, and 77.8% for arboreal. Most misclassifications were with arboreal and terrestrial taxa. Their skeletal proportions were more variable but nevertheless compatible with these two modes of life, contrary to the other modes. The four Balearic representatives of glirids (*i.e. Hypnomys morpheus*, and the three *E. quercinus* from Formentera, Mallorca and Menorca) were included as ungrouped cases in the classification phases of the analysis. Using this procedure, *H. morpheus* was first classified as arboreal (probability *a posteriori*: p = 74.6% - see Table S7) and should be secondly considered as semifossorial (p = 22%). The situation appeared to be more complex for the three Balearic *E. quercinus* because two were classified as arboreal (Formentera, p = 49.2%; Menorca, p = 69.7%) and one as terrestrial (Mallorca, p = 70.8%). As a matter of fact, it was noticed that both terrestrial and arboreal groups showed the lowest percentage of correct classification. Mahalanobis distances indicated that the morphological features of *Hypnomys* were very close to that of arboreal and semifossorial groups (d [*Hypnomys*-arboreal]  = 19, and d [*Hypnomys*-semifossorial]  = 21.4 – see Table S8).

### Body shape

After the reconstruction of Mills (1976)[24], the general body shape of *Hypnomys morpheus* is that of a heavily built dormouse. Our results agree with this approach. From the previous sections it is clear that *Hypnomys morpheus* was larger and displayed different body proportions than its closest living relative, *Eliomys quercinus.* In relation to the length of the skull, some long bones of the limbs were longer in *Hypnomys morpheus* than in *Eliomys quercinus*, mainly the zygopodium bones and the pelvis. Consequently, the estimates for body weight of *Hypnomys* based on skull and dental measurements should be considered as underestimations.

The increase of body size is a clear trend in the *Hypnomys* evolution, identified long ago [23], [24], [40]. Unfortunately nothing is known about bone ratios in the earliest *Eliomys* (i.e., *E. truci* and closest relatives). Thus, comparisons had to be made with recent *E. quercinus*. If *Hypnomys* had a proportionally reduced head or a proportionally increased length of limb bones, a classical measurement as the ratio head/body length cannot be measured accurately without using some reference of the body trunk. We excluded the direct comparison with waist bones, because the main waist bones (scapula, clavicle and pelvis) are directly related to locomotion, and their size is related to the limb bone size.

To calculate the ratio between head and body length in a *Hypnomys* species we used one of the two skeletons found in Cova des Pas de Vallgornera. This ratio was compared with that of recent populations of *Eliomys quercinus* (see Table S7). In comparison to body length, the head became increasingly smaller when *Eliomys* became elder. The specimen of *Hypnomys* from Vallgornera had a proportionally slightly smaller head than the living garden dormice. Consequently, it is the skull of *Hypnomys* that appears as relatively reduced. Thus, again we can conclude than our initial estimation of body mass should be considered as an underestimate.

## Discussion

Mills (1976)[24] stated that *Hypnomys* was a robust and probably powerfully muscled dormouse. This author studied non-associated material and established that the relative lengths of the antebrachium and tibia compared to the humerus and femur suggest that *Hypnomys* was less cursorial than other dormice.

Tracks and trackways of *Hypnomys* were found on aeolianites in two Menorcan deposits [41], [42]. Data yielded from the Cova de Sa Duna deposit (Alaior), and the lack of tail tracks was taken as evidence that *Hypnomys* did not drag the tail [42], contrary to [24].

The results obtained from the statistical analyses showed a complex situation that does not drive to clear-cut conclusions. That situation cannot be understood unless is first considered the fact that the individuals integrate the selective effects of the many components of the environment, and second, that are unknown the early conditions of any insular evolution. In our analysis, the three living Balearic Garden Dormice depicted an original case of differentiation. One of them (Formentera population) showed a more terrestrial way of life. *Eliomys* is known to inhabit macchias, but also lives on the floor of rocky areas with blocks of rocks in vegetated areas and it can be expected that populations may become more specialized in one or another component of this bivalent mode of life (*i.e.* arboreal *vs* terrestrial). The difference between the *Eliomys* from Formentera and Menorca, and the *Eliomys* from Mallorca already illustrated insular divergences involving the whole morphology of the cranial and post-cranial skeleton. *Hypnomys* illustrates a different case. The results showed that *Hypnomys* was able to climb in the tree as well as to live on the ground. However, we should note from the classification procedure the very low percentage observed for *Hypnomys* as a typical terrestrial rodent (probability *a posteriori*: p = 0.03%!). Thus, we have to take into consideration that *Hypnomys* was able to dig. This hypothesis is in agreement with the fact that all the specimens of *H. morpheus* showed a high number of pits and large pits [25]. Such a high frequency of coarse features could indicate the intake of grit into the diet [43]. Contrary to *Eliomys*, *Hypnomys* might have developed a more fossorial behaviour. As *Hypnomys* lived under a Mediterranean climate and flora, it would be possible to consider that it may have lived on some vegetal matter provided by geophytes, at least when conditions were extreme.

Little is known about the environment which *Hypnomys* inhabited. Climate and vegetation in the Western Mediterranean during Early Pliocene was markedly subtropical [44], [45]. It is assumed that since the late Pliocene the subtropical vegetation evolved to a Mediterranean one, with a more arid character than the previous one [46]. In some of the interglacial periods the climate of the Balearic Islands showed a wide seasonal range of temperature, moisture and precipitation, including recorded periods with a mean annual temperature about 2°C higher than today, with very dry moisture regimes, limited plant biomass [47], [48], and important sea-level rises [49], [50]. The available data on the Late Pleistocene-Holocene vegetation from Mallorca and Menorca revealed the presence of abundant plant taxa with scoriaceous leaves and containing toxic alkaloids (e.g., *Juniperus*, *Buxus*, *Ephedra*)[51], [52]. Nevertheless, it still remains unclear how this vegetation, supposedly modelled by climate and *Myotragus*, could have influenced the evolution of *Hypnomys*.

It is difficult to find parallels to the *Hypnomys* body shape and proportions. We have been unable to find clear analogies to the zygopodium elongation in living Sciurognaths. This elongation suggests a peculiar way of locomotion, which should have been unusual for Sciurognaths. Similar, but not identical, body proportions are present in the fossil insular rat *Canariomys tamarani* from Gran Canaria [53], but not in *Canariomys bravoi* from Tenerife. *C. tamarani* also displays a proportionally longer radius and tibia and a wider distal humerus. The bone proportions of *C. tamarani* have been interpreted as an adaptation to a more terrestrial life style with greater cursorial and fossorial abilities than in *C. bravoi* [53]. It is currently unknown whether the limb proportions observed in *Hypnomys* and *C. tamarani* are shared by other insular rodents, and are consequently a result of an insular evolutionary trend.

Isolation on islands is a well-known factor to induce evolutionary change and a rather frequent event is the origination of giant forms in small mammals like rodents, and the study of the skeleton not only provides a good illustration of the increase in size, but also of changes in shape [54]–[57]. The singularity of *Hypnomys* in relation to *Eliomys* illustrates that aside size, differences in shape resulted of an adaptive process in relation with several aspects of the lifestyle that included the modes of locomotion, of foraging, and of food processing. Because of the lack of competitors, *Hypnomys* was able to become more a burrower and thus to diverge from its ancestral condition, presumed to be that of *Eliomys* (*i.e.* forest and floor of rocky areas).

Within the 30 living species of Gliridae, only the West African *Graphiurus crassicaudatus* has a tail as short (relative tail length 65.7%)[58] as in *Hypnomys* (64.7%). Species with moderately short tails are *Graphiurus surdus* (72.9%), and *Myomys roachi* (77.3%)[59]. Other glirids have relative tail lengths over 80%. *Myomys roachi* lives on the ground, with no morphological adaptations for a scansorial or fossorial life style [59]. The ecology of *Graphiurus crassicaudatus* is poorly known. It would be promising to study skeletons of these rare species in the future.

Finally, we want to emphasize on opposite trends recorded in the evolution of two herbivorous mammals which inhabited the Gymnesic Islands until the human arrival. *Myotragus balearicus* was a dwarfed ruminant that displayed a reduction of the distal part of limbs, with an extremely reduced ability for running [9]. *Hypnomys morpheus* was an enlarged dormouse derived from an *Eliomys* ancestor, with elongated limbs, and perhaps an increased cursorial ability.

## Supporting Information

Appendix S1 **List of extant and extinct rodent skeletons measured in this paper.**
(DOC)

Appendix S2 **Postcranial indexes used in this paper (obtained from [33] and [60]).**
(DOC)

Table S1 **Body weight estimates of *H. morpheus* from Cova des Coral·loides.**
(DOC)

Table S2 ***Eliomys* versus *Hypnomys* skull indexes.**
(DOC)

Table S3 ***Eliomys* versus *Hypnomys* robustness indexes (limb bones).**
(DOC)

Table S4 ***Eliomys* versus *Hypnomys* morphofunctional indexes (limb bones).**
(DOC)

Table S5 **Limb bone proportion indexes and comparison with ranges (95% interval) for terrestrial, arboreal and semifossorial rodents (according to [33]).**
(DOC)

Table S6 **Limb versus skull indexes in *Eliomys* and *Hypnomys*.**
(DOC)

Table S7 **Probability a posteriori (%) for the locomotor habit of Balearic dormice.**
(DOC)

Table S8 **Mahalanobis distances between the morphological proportions of *Eliomys quercinus* and *Hypnomys morpheus* vs different types of locomotor habit.**
(DOC)
