# Supplementary material for: Body Shape and Life Style of the Extinct Balearic Dormouse Hypnomys (Rodentia, Gliridae): New Evidence from the Study of Associated Skeletons
Source: PLoS One. 2010 Dec 31;5(12):e15817. doi: 10.1371/journal.pone.0015817 (PMC3013122; doi:10.1371/journal.pone.0015817)
Supplement: Appendix S1 — List of extant and extinct rodent skeletons measured in this paper. (DOC) [file pone.0015817.s001.doc]

| **Species** | **Subsp.** | **Collection number** | **Island** | **Description** |
| --- | --- | --- | --- | --- |
| *Eliomys quercinus* | *ophiusae* | IMEDEA 7343 | Formentera | Complete skeleton |
| *Eliomys quercinus* | *ophiusae* | IMEDEA 7341 | Formentera | Complete skeleton |
| *Eliomys quercinus* | *ophiusae* | IMEDEA 7340 | Formentera | Complete skeleton |
| *Eliomys quercinus* | *ophiusae* | IMEDEA 7345 | Formentera | Complete skeleton |
| *Eliomys quercinus* | *quercinus* | IMEDEA 7335 | Mallorca | Complete skeleton |
| *Eliomys quercinus* | *quercinus* | IMEDEA 7336 | Mallorca | Complete skeleton |
| *Eliomys quercinus* | *quercinus* | IMEDEA 7430 | Mallorca | Complete skeleton |
| *Eliomys quercinus* | *quercinus* | IMEDEA 7315 | Mallorca | Complete skeleton |
| *Eliomys quercinus* | *gymnesicus* | IMEDEA 7406 | Menorca | Complete skeleton |
| *Eliomys quercinus* | *gymnesicus* | IMEDEA 7451 | Menorca | Complete skeleton |
| *Eliomys quercinus* | *gymnesicus* | IMEDEA 13307 | Menorca | Complete skeleton |
| *Hypnomys morpheus* |  | CDC 2 | Mallorca | Complete skeleton |
| *Hypnomys morpheus* |  | CDC 10 | Mallorca | Partial skeleton |
| *Hypnomys morpheus* |  | CDC 27 | Mallorca | Partial skeleton |
| *Hypnomys morpheus* |  | CDC 39 | Mallorca | Partial skeleton |
| *Hypnomys morpheus* |  | CDC 40 | Mallorca | Partial skeleton |
