# Supplementary material for: Body Shape and Life Style of the Extinct Balearic Dormouse Hypnomys (Rodentia, Gliridae): New Evidence from the Study of Associated Skeletons
Source: PLoS One. 2010 Dec 31;5(12):e15817. doi: 10.1371/journal.pone.0015817 (PMC3013122; doi:10.1371/journal.pone.0015817)
Supplement: Appendix S2 — Postcranial indexes used in this paper (obtained from [33] and [60]). (DOC) [file pone.0015817.s002.doc]

***Shoulder moment index (SMI):*** Deltopectoral crest length divided by functional length of the humerus (DPCL/HL). Indicates mechanical advantage of the deltoid and pectoral muscles acting across the shoulder joint.

***Brachial index (BI):*** Functional length of the radius divided by functional length of the humerus (RL/HL). Indicates relative proportions of proximal and distal elements of the forelimb.

***Humeral robustness index (HRI):*** Mediolateral diameter of humerus divided by functional length of the humerus (HMLD/HL). Indicates robustness of the humerus and its ability to resist bending and shearing stresses.

***Humeral epicondylar index (HEB):*** Epicondylar breadth of humerus divided by functional length of the humerus (HEB/HL). Indicates relative area available for the origins of the forearm flexors, pronators, and supinators.

***Olecranon length index (OLI):*** Olecranon process length divided by functional length of the ulna (UOL/FUL). Indicates relative mechanical advantage of the triceps brachii and dorsoepitrochlearis muscles used in elbow extension. This is identical to the index of fossorial ability used by Hildebrand (1985).

***Ulnar robustness index (URI):*** Mediolateral diameter of ulna divided by functional length of the ulna (UMLD/FUL). Indicates robustness of the ulna and its ability to resist bending and shearing stresses, and relative area available for the origin and insertion of forearm and manus flexors, pronators, and supinators.

***Crural index (CI):*** Functional length of the tibia divided by functional length of the femur (TL/FL). Indicates relative proportions of proximal and distal elements of the hind limb.

***Femoral robustness index (FRI):*** Anteroposterior diameter of femur divided by functional length of the femur (FAPD/FL). Indicates robustness of the femur and its ability to resist bending and shearing stresses (AP diameter is used due to transverse expansion of the femora in semiaquatic rodents).

***Gluteal index (GI)*** Length of distal extension of the greater trochanter of the femur divided by functional length of the femur (FGT/FL). Indicates relative mechanical advantage of the gluteal muscles used in retraction of the femur.

***Femoral epicondylar index (FEB):*** Epicondylar breadth of femur divided by the functional length of the femur (FEB/FL). Indicates relative area available for the origins of the gastrocnemius and soleus muscles used in extension of the knee and plantar-flexion of the pes.

***Tibial robustness index (TRI):*** Mediolateral diameter of tibia divided by functional length of the tibia (TMLD/TL). Indicates robustness of the tibia and its ability to resist bending and shearing stresses.

***Tibial spine index (TSI):*** Length of distal extension of the tibial tuberosity (spine) divided by functional length of the tibia (TSL/TL). Indicates relative mechanical advantage of the hamstrings and biceps femoris muscles acting across the knee and hip joints.

***Intermembral index (IM):*** Functional lengths of the humerus and radius divided by lengths of the femur and tibia [(HL + RL)/(FL + TL)]. Indicates the length of the forelimb relative to the hind limb.
