# Supplementary material for: Body Shape and Life Style of the Extinct Balearic Dormouse Hypnomys (Rodentia, Gliridae): New Evidence from the Study of Associated Skeletons
Source: PLoS One. 2010 Dec 31;5(12):e15817. doi: 10.1371/journal.pone.0015817 (PMC3013122; doi:10.1371/journal.pone.0015817)
Supplement: Table S1 — Body weight estimates of H. morpheus from Cova des Coral·loides. (DOC) [file pone.0015817.s003.doc]

**Table S1.** Body weight estimates of *H. morpheus* from Cova des Coral·loides.

| **Measurement (mm)** | **Source** | **Intercept** | **Slope** | ***H. morpheus***  **Estimation (g)** | ***E. quercinus***  **7335 (74g)** |
| --- | --- | --- | --- | --- | --- |
| **CBL** | Calculated from [35]* | -7.8744* | 3.4701* | 214* | 64* |
| LTRL | [22] (Muroidea<500 g)* | -0.6196* | 2.7020* | 123* | 48* |
| LTRL | [22] (Muroidea<5 kg)* | -0.6449* | 2.7160* | 124* | 48* |
| **RTRA** | [22] (Muroidea<500 g)* | 0.9320* | 1.5199* | 184* | 77* |
| **RTRA** | [22] (Muroidea<5 kg)* | 1.0516* | 1.4516* | 173* | 75* |
| **IMD (LI in ref)** | [19]* | 2.71* | 2.94* | 260* | 66* |
| FW | [36] (recalculated from [18]) | -4.18 | 3.02 | 81 | 23 |
| IW | [36] (recalculated from [18]) | -2.16 | 2.52 | 366 | 144 |
| ZL | [36] (recalculated from [18]) | -5.13 | 3.35 | 148 | 49 |
| BCL | [36] (recalculated from [18]) | -4.9 | 3.82 | 513 | 187 |
| RoL (RL in reference) | [36] (recalculated from [18]) | -5.34 | 3.32 | 151 | 43 |
| DL | [36] (recalculated from [18]) | -4.61 | 3.53 | 117 | 24 |
| STL (TL in reference) | [36] (recalculated from [18]) | -6.68 | 3.56 | 172 | 51 |
| UTL (UTRW in reference) | [36] | -0.0380 | 2.7 | 297 | 100 |
| Upper T | [36] | 2.08 | 2.45 | 507 | 220 |
| **CBL (Skull in reference)** | [36] | -3.33 | 3.49 | 284 | 84 |
| m1 surface | [37]* (Obtained from [19]) | 2.0875* | 1.7548* | 92* | 41* |
| AP Diameter (Humerus) OLS | [38] | -1.467 | 2.484 | 402 | 170 |
| AP Diameter (Humerus) RMA | [38] | -1.486 | 2.516 | 397 | 166 |

Estimates based on log and ln (*) transformations of allometric models obtained from literature. An individual of *E. quercinus* (IMEDEA 7335) with known body weight (last column) has been used to test the reliability of estimations (bold lettered estimates indicate best proxies). **AP Diameter (Humerus) OLS**: Anterio-Posterior Humeral Diameter at 35% from the distal articular surface (Ordinary Least Squares Regression); **AP Diameter (Humerus) RMA**: Anterio-Posterior Humeral Diameter at 35% from the distal articular surface (Reduced Major Axis Regression); **BCL**: Basicranial Length; **CBL**: Condylobasal Length; **DL**: Diastema Length; **FW**: Frontal Width; **IMD**: Incisor Mesio Distal Diameter measured at wear facet level; **IW**: Incisors Width; **LTRL**: Lower Toothrow Length; **RoL**: Rostral Length; **RTRA**: Rectangular Lower Toothrow Area; **STL**: Skull Total Length; **UTL**: Upper Toothrow Length; **Upper T**: Upper Incisor Transverse Diameter; **ZL**: Zygomatic Arch Length.
