# Supplementary material for: Body Shape and Life Style of the Extinct Balearic Dormouse Hypnomys (Rodentia, Gliridae): New Evidence from the Study of Associated Skeletons
Source: PLoS One. 2010 Dec 31;5(12):e15817. doi: 10.1371/journal.pone.0015817 (PMC3013122; doi:10.1371/journal.pone.0015817)
Supplement: Table S2 — Eliomys versus Hypnomys skull indexes. (DOC) [file pone.0015817.s004.doc]

**Table S2.** *Eliomys* versus *Hypnomys* skull indexes.

|  |  | ***E. quercinus* MA** | | | ***E. quercinus* ME** | | | ***E. quercinus* FO** | | | ***H. morpheus*** | |
| --- | --- | --- | --- | --- | --- | --- | --- | --- | --- | --- | --- | --- |
|  |  | **N** | **X** | **Range** | **N** | **X** | **Range** | **n** | **X** | **Range** | **n** | **X** |
| *Rostrum Length* | |  |  |  |  |  |  |  |  |  |  |  |
|  | RoL/CBL | 15 | 47.68 | 46.65-48.76 | 22 | 47.67 | 46.20-49.52 | 10 | 49.12 | 48.21-49.75 | 1 | 50.54 |
|  | NL/CBL | 15 | 39.08 | 37.29-40.82 | 22 | 38.53 | 34.85-40.09 | 10 | 42.54 | 41.12-44.18 | 1 | 37.02 |
|  | DL/CBL | 15 | 23.30 | 22.05-24.61 | 22 | 23.10 | 21.62-24.76 | 10 | 24.76 | 24.09-25.64 | 1 | 25.24 |
|  | FIL/CBL | 15 | 14.99 | 13.50-15.99 | 22 | 14.49 | 13.41-15.71 | 10 | 15.96 | 14.07-17.34 | 1 | 15.95 |
| *Braincase Length* | |  |  |  |  |  |  |  |  |  |  |  |
|  | BNL/CBL | 15 | 52.41 | 50.78-53.51 | 22 | 52.26 | 49.37-54.21 | 10 | 50.69 | 49.87-51.65 | 1 | 49.46 |
| *Rostrum Width* | |  |  |  |  |  |  |  |  |  |  |  |
|  | RW/CBL | 15 | 20.71 | 19.42-21.78 | 22 | 20.26 | 19.02-21.70 | 10 | 20.47 | 20.02-21.39 | 1 | 20.54 |
|  | IOW/CBL | 14 | 15.31 | 14.33-16.36 | 22 | 15.26 | 14.37-16.40 | 10 | 13.68 | 13.21-14.20 | 1 | 16.20 |
|  | ZW/CBL | 14 | 62.32 | 61.04-63.05 | 21 | 62.14 | 59.49-64.95 | 10 | 57.52 | 54.87-59.24 | 1 | 62.69 |
|  | M2-M2/CBL | 15 | 22.23 | 20.25-24.18 | 21 | 22.46 | 20.09-24.41 | 10 | 21.38 | 20.55-22.39 | 1 | 23.73 |
| *Braincase Width* | |  |  |  |  |  |  |  |  |  |  |  |
|  | MW/CBL | 15 | 40.29 | 38.71-41.76 | 22 | 38.95 | 36.87-41.40 | 10 | 39.07 | 37.98-40.38 | 1 | 35.48 |
|  | BNW/CBL | 3 | 49.11 | 47.34-50.48 | 2 | 47.55 | 47.32-47.78 | 4 | 42.79 | 41.76-44.44 | 1 | 44.13 |
|  | BNW/BNL | 3 | 96.03 | 92.91-98.30 | 2 | 92.22 | 92,20-92.24 | 4 | 83.36 | 82.12-85.40 | 1 | 89.23 |
| *Braincase Height* | |  |  |  |  |  |  |  |  |  |  |  |
|  | BNH/CBL | 14 | 32.41 | 30.72-34.06 | 22 | 32.24 | 29.91-34.67 | 10 | 28.89 | 27.97-29.66 | 1 | 28.70 |
| *Tympanic Bulla* | |  |  |  |  |  |  |  |  |  |  |  |
|  | TBL/CBL | 15 | 31.09 | 29.75-32.72 | 22 | 30.28 | 28.99-31.25 | 10 | 29.65 | 28.24-33.08 | 1 | 27.32 |
|  | TBW/CBL | 15 | 19.05 | 18.41-19.93 | 22 | 18.74 | 17.37-19.75 | 10 | 17.06 | 16.00-17.93 | 1 | 18.99 |
|  | TBw/CBL | 15 | 15.30 | 14.48-16.47 | 22 | 14.74 | 12.99-15.74 | 10 | 13.65 | 13.21-14.09 | 1 | 15.80 |
|  | TBL/BNL | 15 | 59.35 | 56.48-62.58 | 22 | 57.96 | 54.86-62.10 | 10 | 58.49 | 54.68-64.82 | 1 | 55.23 |
| *Upper Toothrow* | |  |  |  |  |  |  |  |  |  |  |  |
|  | UTL/CBL | 14 | 16.74 | 16.08-18.29 | 20 | 16.77 | 16.10-18.43 | 4 | 15.91 | 15.57-16.32 | 1 | 17.83 |
|  | UTL/RoL | 14 | 35.07 | 33.66-37.50 | 25 | 35.04 | 33.44-37.22 | 4 | 32.38 | 31.98-32.81 | 1 | 35.28 |
| *Mandible* | |  |  |  |  |  |  |  |  |  |  |  |
|  | ML/CBL | 15 | 57.18 | 55.18-59.43 | 21 | 57.45 | 55.54-59.94 | 10 | 57.63 | 56.41-59.37 | 1 | 57.98 |

**BNH**: Braincase Height; **BNL**: Braincase Length; **BNW**: Braincase Width; **CBL**: Condylobasal Length; **DL**: Diastema Length; **FIL**: Foramina Incisivi Length; **IOW**: Interorbitary Width; **M2-M2**: Breadth between labial margins of M2; **ML**: Mandibular Length; **MW**: Mastoid Width; **NL**: Nasal Length; **RoL**: Rostral Length; **RW**: Rostral Width; **TBL**: Tympanic Bulla Length; **TBW**: Tympanic Bulla Maximum Width; **TBw**: Tympanic Bulla Mimimun Width; **UTL**: Upper Toothrow Length; **ZW**: Zygomatic Width. **MA**: Mallorca; **ME**: Menorca; **FO**: Formentera.
