# Supplementary material for: Body Shape and Life Style of the Extinct Balearic Dormouse Hypnomys (Rodentia, Gliridae): New Evidence from the Study of Associated Skeletons
Source: PLoS One. 2010 Dec 31;5(12):e15817. doi: 10.1371/journal.pone.0015817 (PMC3013122; doi:10.1371/journal.pone.0015817)
Supplement: Table S3 — Eliomys versus Hypnomys robustness indexes (limb bones). (DOC) [file pone.0015817.s005.doc]

**Table S3.** *Eliomys* versus *Hypnomys* robustness indexes (limb bones).

|  | **HRI** | | | **URI** | | | **FRI** | | | **TRI** | | |
| --- | --- | --- | --- | --- | --- | --- | --- | --- | --- | --- | --- | --- |
|  | **N** | **X** | **Range** | **n** | **X** | **Range** | **n** | **X** | **Range** | **n** | **X** | **Range** |
| *E. quer.* FO | 4 | 0.103 | 0.098-0.110 | 4 | 0.043 | 0.039-0.049 | 4 | 0.082 | 0.078-0.084 | 3 | 0.066 | 0.062-0.068 |
| *E. quer.* MA | 4 | 0.095 | 0.089-0.097 | 4 | 0.036 | 0.033-0.039 | 3 | 0.073 | 0.070-0.076 | 4 | 0.055 | 0.051-0.058 |
| *E. quer.* ME | 3 | 0.091 | 0.090-0.092 | 3 | 0.036 | 0.035-0.038 | 3 | 0.071 | 0.066-0.075 | 3 | 0.057 | 0.053-0.059 |
| *Hypnomys* | 4 | 0.109 | 0.106-0.116 | 4 | 0.041 | 0.038-0.042 | 3 | 0.098 | 0.088-0.112 | 3 | 0.060 | 0.059-0.063 |

**HRI**: Humeral Robustness Index; **URI**: Ulnar Robustness Index; **FRI**: Femoral Robustness Index; **TRI**: Tibial Robustness Index; ***E. quer.***: *Eliomys quercinus*; **MA**: Mallorca; **ME**: Menorca; **FO**: Formentera.
