# Supplementary material for: Body Shape and Life Style of the Extinct Balearic Dormouse Hypnomys (Rodentia, Gliridae): New Evidence from the Study of Associated Skeletons
Source: PLoS One. 2010 Dec 31;5(12):e15817. doi: 10.1371/journal.pone.0015817 (PMC3013122; doi:10.1371/journal.pone.0015817)
Supplement: Table S4 — Eliomys versus Hypnomys morphofunctional indexes (limb bones). (DOC) [file pone.0015817.s006.doc]

**Table S4.** *Eliomys* versus *Hypnomys* morphofunctional indexes (limb bones).

|  | **SMI** | | | **HEB** | | | **OLI** | | | **GI** | | | **FEB** | | | **TSI** | | |
| --- | --- | --- | --- | --- | --- | --- | --- | --- | --- | --- | --- | --- | --- | --- | --- | --- | --- | --- |
|  | **n** | **X** | **Range** | **N** | **X** | **Range** | **n** | **X** | **Range** | **n** | **X** | **Range** | **n** | **X** | **Range** | **n** | **X** | **Range** |
| *E. q.* FO | 4 | 0.457 | 0.432-0.472 | 4 | 0.260 | 0.250-0.276 | 4 | 0.138 | 0.129-0.143 | 4 | 0.120 | 0.113-0.127 | 3 | 0.187 | 0.177-0.183 | 3 | 0.390 | 0.341-0.414 |
| *E. q.* MA | 4 | 0.420 | 0.406-0.431 | 4 | 0.256 | 0.239-0.292 | 4 | 0.126 | 0.119-0.130 | 3 | 0.108 | 0.099-0.118 | 3 | 0.183 | 0.172-0.193 | 4 | 0.362 | 0.308-0.400 |
| *E. q.* ME | 3 | 0.417 | 0.415-0.420 | 3 | 0.256 | 0.252-0.263 | 3 | 0.136 | 0.120-0.156 | 3 | 0.109 | 0.103-0.114 | 3 | 0.179 | 0.172-0.183 | 3 | 0.384 | 0.378-0.389 |
| *Hypnomys* | 4 | 0.464 | 0.447-0.478 | 4 | 0.316 | 0.294-0.339 | 4 | 0.142 | 0.134-0.151 | 3 | 0.118 | 0.114-0.123 | 3 | 0.218 | 0.203-0.229 | 3 | 0.397 | 0.389-0.402 |

**SMI**: Shoulder Moment Index; **HEB**: Humeral Epicondylar Index; **OLI**: Olecranon Length Index; **GI**: Gluteal Index; **FEB**: Femoral Epicondylar Index; **TSI**: Tibial Spine Index; ***E. q.***: *Eliomys quercinus*; **MA**: Mallorca; **ME**: Menorca; **FO**: Formentera.
