# Supplementary material for: Body Shape and Life Style of the Extinct Balearic Dormouse Hypnomys (Rodentia, Gliridae): New Evidence from the Study of Associated Skeletons
Source: PLoS One. 2010 Dec 31;5(12):e15817. doi: 10.1371/journal.pone.0015817 (PMC3013122; doi:10.1371/journal.pone.0015817)
Supplement: Table S5 — Limb bone proportion indexes and comparison with ranges (95% interval) for terrestrial, arboreal and semifossorial rodents (according to [33]). (DOC) [file pone.0015817.s007.doc]

**Table S5.** Limb bone proportion indexes and comparison with ranges (95% interval) for terrestrial, arboreal and semifossorial rodents (according to [33]).

|  | **BI** | | | **CI** | | | **IM** | | |
| --- | --- | --- | --- | --- | --- | --- | --- | --- | --- |
|  | **n** | **X** | **Range** | **n** | **X** | **Range** | **n** | **X** | **Range** |
| *E. quer.* FO | 4 | 0.981 | 0.954-1.018 | 3 | 1.136 | 1.110 -1.176 | 3 | 0.732 | 0.720-0.740 |
| *E. quer.* MA | 4 | 0.977 | 0.958-1.003 | 3 | 1.159 | 1.126-1.220 | 3 | 0.723 | 0.718-0.731 |
| *E. quer.* ME | 3 | 0.966 | 0.917-1.010 | 3 | 1.127 | 1.089-1.152 | 3 | 0.730 | 0.719-0.740 |
| *Hypnomys* | 2 | 1.151 | 1.110-1.192 | 2 | 1.265 | 1.201-1.330 | 1 | 0.704 |  |
| Terrestrial |  |  | 0.85-1.14 |  |  | 1.06-1.37 |  |  | 0.60-0.871 |
| Arboreal |  |  | 0.81-1.05 |  |  | 0.911-1.227 |  |  | 0.656-0.944 |
| Semifossorial |  |  | 0.739-1.109 |  |  | 0.911-1.267 |  |  | 0.605-0.887 |

**BI** : Brachial Index; **CI** : Crural Index; **IM** : Intermembral Index; ***E. quer.***: *Eliomys quercinus*; **MA**: Mallorca; **ME**: Menorca; **FO**: Formentera.
