# Supplementary material for: Body Shape and Life Style of the Extinct Balearic Dormouse Hypnomys (Rodentia, Gliridae): New Evidence from the Study of Associated Skeletons
Source: PLoS One. 2010 Dec 31;5(12):e15817. doi: 10.1371/journal.pone.0015817 (PMC3013122; doi:10.1371/journal.pone.0015817)
Supplement: Table S6 — Limb versus skull indexes in Eliomys and Hypnomys. (DOC) [file pone.0015817.s008.doc]

**Table S6.** Limb versus skull indexes in *Eliomys* and *Hypnomys*.

|  | **HL/CBL** | | | **RL/CBL** | | | **FUL/CBL** | | | **FL/CBL** | | | **TL/CBL** | | | **SL/CBL** | | | **PL/CBL** | | |
| --- | --- | --- | --- | --- | --- | --- | --- | --- | --- | --- | --- | --- | --- | --- | --- | --- | --- | --- | --- | --- | --- |
|  | **n** | **X** | **Range** | **n** | **X** | **Range** | **n** | **X** | **Range** | **n** | **X** | **Range** | **n** | **X** | **Range** | **n** | **X** | **Range** | **n** | **X** | **Range** |
| *E. q.* FO | 4 | 0.57 | 0.55-0.59 | 4 | 0.56 | 0.56-0.57 | 4 | 0.62 | 0.61-0.62 | 4 | 0.73 | 0.68-0.76 | 3 | 0.83 | 0.81-0.84 | 3 | 0.46 | 0.45-0.48 | 4 | 0.74 | 0.71-0.75 |
| *E. q.* MA | 3 | 0.58 | 0.54-0.6 | 3 | 0.57 | 0.54-0.58 | 3 | 0.62 | 0.59-0.65 | 3 | 0.74 | 0.68-0.77 | 3 | 0.85 | 0.83-0.87 | 2 | 0.45 | 0.42-0.48 | 3 | 0.73 | 0.67-0.76 |
| *E. q.* ME | 2 | 0.58 | 0.57-0.6 | 2 | 0.58 | 0.58-0.58 | 2 | 0.59 | 0.57-0.62 | 2 | 0.76 | 0.75-0.78 | 2 | 0.84 | 0.84-0.85 | 2 | 0.47 | 0.47-0.48 | 2 | 0.73 | 0.72-0.74 |
| ***Hypnomys*** | 1 | 0.56 |  | 1 | 0.67 |  | 1 | 0.72 |  | 1 | 0.8 |  | 1 | 0.96 |  | 1 | 0.46 |  | 1 | 0.81 |  |

**CBL**: Condylobasal Length; **FL**: Femur Length; **FUL**: Ulna Functional Length; **HL**: Humerus Length; **PL**: Pelvis Length; **RL**: Radius Length; **SL**: Scapula Length; **TL**: Tibia Length; ***E. q.***: *Eliomys quercinus*: **MA**: Mallorca; **ME**: Menorca; **FO**: Formentera.
