# Supplementary material for: Body Shape and Life Style of the Extinct Balearic Dormouse Hypnomys (Rodentia, Gliridae): New Evidence from the Study of Associated Skeletons
Source: PLoS One. 2010 Dec 31;5(12):e15817. doi: 10.1371/journal.pone.0015817 (PMC3013122; doi:10.1371/journal.pone.0015817)
Supplement: Table S7 — Probability a posteriori (%) for the locomotor habit of Balearic dormice. (DOC) [file pone.0015817.s009.doc]

**Table S7.** Probability a posteriori (%) for the locomotor habit of Balearic dormice.

|  |  | **Ricochetal** | **Gliding** | **Semifossorial** | **Terrestrial** | **Semiaquatic** | **Arboreal** | **Fossorial** |
| --- | --- | --- | --- | --- | --- | --- | --- | --- |
| ***Eliomys quercinus*** | **Formentera** | 0.00 | 0.00 | 12.8 | 38 | 0.00 | **49.2** | 0.00 |
| ***Eliomys quercinus*** | **Mallorca** | 0.00 | 0.00 | 1.2 | **70.8** | 0.00 | 27.9 | 0.00 |
| ***Eliomys quercinus*** | **Menorca** | 0.00 | 0.00 | 6.4 | 23.9 | 0.00 | **69.7** | 0.00 |
| ***Hypnomys morpheus*** | **Coral·loides** | 0.00 | 0.00 | 22 | 3.3 | 0.00 | **74.6** | 0.00 |
