# Supplementary material for: Body Shape and Life Style of the Extinct Balearic Dormouse Hypnomys (Rodentia, Gliridae): New Evidence from the Study of Associated Skeletons
Source: PLoS One. 2010 Dec 31;5(12):e15817. doi: 10.1371/journal.pone.0015817 (PMC3013122; doi:10.1371/journal.pone.0015817)
Supplement: Table S8 — Mahalanobis distances between the morphological proportions of Eliomys quercinus and Hypnomys morpheus vs different types of locomotor habit. (DOC) [file pone.0015817.s010.doc]

**Table S8.** Mahalanobis distances between the morphological proportions of *Eliomys quercinus* and *Hypnomys morpheus* vs different types of locomotor habit.

|  |  | **Ricochetal** | **Gliding** | **Semifossorial** | **Terrestrial** | **Semiaquatic** | **Arboreal** | **Fossorial** |
| --- | --- | --- | --- | --- | --- | --- | --- | --- |
| ***Eliomys quercinus*** | **Formentera** | 64.3 | 39.2 | 13.1 | 11.8 | 33.7 | **10.4** | 33.2 |
| ***Eliomys quercinus*** | **Mallorca** | 82.9 | 32.2 | 16.4 | **9.2** | 30.7 | 10.1 | 35.6 |
| ***Eliomys quercinus*** | **Menorca** | 75 | 25.4 | 10.6 | 8.8 | 32.3 | **5.8** | 31 |
| ***Hypnomys morpheus*** | **Coral·loides** | 52.8 | 53.4 | 21.4 | 26 | 39.7 | **19** | 40.4 |
